# Changes in strength and power performance and serum hormone concentrations during 12 weeks of task‐specific or strength training in conscripts

**DOI:** 10.14814/phy2.14422

**Published:** 2020-05-06

**Authors:** Tommi Ojanen, Heikki Kyröläinen, Elena Kozharskaya, Keijo Häkkinen

**Affiliations:** ^1^ Finnish Defence Research Agency Finnish Defence Forces Järvenpää Finland; ^2^ Biology of Physical Activity University of Jyväskylä Jyväskylä Finland; ^3^ National Defence University Helsinki Finland

**Keywords:** hormonal, Physical training, power, soldiers, strength

## Abstract

The purpose of this study was to investigate the effects of two different training programs on strength and power performance and serum hormone concentrations. A total of 104 male soldiers volunteered and took part in the 12‐week training period with baseline, mid‐, and post‐measurements of body composition, muscle strength, lower and upper body power, and blood samples to determine serum hormone concentrations. The mean (±*SD*) age of subjects was 20 ± 1 years, height 180 ± 6 cm and body mass 72.4 ± 8.8 kg. The subjects were divided into three different training groups: soldier task‐specific training (TS), strength training (ST), and control (CON). Each group had a total of 18 training sessions during the 12‐week study. In the muscle strength tests, most improvements could be observed in the TS and ST groups, especially, during the first weeks of the training period. Maximal isometric leg extension force increased significantly by 7.9 ± 12.2% (*p* < .05) in the TS and 7.1 ± 12.6% (*p* < .05) in the ST groups between the PRE and MID, as well as between the PRE and POST measurements by 8.1 ± 12.4% (*p* < .05) in TS and 12.3 ± 15.3% (*p* < .01) in ST. Serum TES concentration increased significantly in TS between the PRE and MID (16.8 ± 33.9%) and PRE and POST (11.2 ± 16.7%) measurements. Serum COR concentrations decreased in TS between the MID and POST (−7.8 ± 10.9%) and PRE and POST (−11.0 ± 14.3%) measurements. Although the differences observed were rather minor in magnitude, training in the TS and ST groups led to greater improvements in muscle strength and power performance compared to the training in the CON group. The development of strength and/or power of the lower and upper body was greater in the TS and ST groups, which is crucial for warfighter's performance. Therefore, it is important to have a structured resistance‐training program during military training to optimize the strength, power, and military‐specific performance.

## Graphical abstract

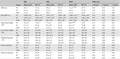

## 1 INTRODUCTION

Modern warfighting requires a high level of physical performance so that soldiers would be capable to successfully fulfill missions in the field (Nindl et al., 2013). It is important to have appropriate strength, endurance, and other physical capabilities to perform different military tasks, such as sprinting, lifting, and carrying load over obstacles and in various terrains (Kraemer & Szivak, 2012; Kyröläinen, Pihlainen, Vaara, Ojanen, & Santtila, 2018; Nindl et al., 2017). However, military training in the field consists mainly of prolonged aerobic physical activity with low training intensities. This kind of daily activity could potentially interfere with both optimal muscle strength and maximal aerobic capacity development (Friedl et al., 2015). Despite this, it is still quite uncommon to have structured and periodized training programs for optimal muscular strength and endurance development during military training (Szivak & Kraemer, 2015).

Physical training and consequent adaptations are influenced by several factors such as age, gender, sleep, nutrition, training history, recovery, as well as by environmental, social, and psychological factors (Kyröläinen et al., 2018). In the military environment, prolonged physical activity with external load, sleep deprivation, and negative fluid and nutrition balance are also obstacles that one must overcome in optimizing physical performance gains (Henning, Park, & Kim, 2011; Nindl et al., 2007). It has been shown that these training and environmental factors can lead to overreaching or even overtraining leading to increased risk of injury occurrence (Booth, Probert, Forbes‐Ewan, & Coad, 2006). It is important to consider these factors when planning and implementing training programs for warfighters.

Currently, military duties require more physical fitness than in the past (Friedl et al., 2015; Knapik, Reynolds, & Harman, 2004). In recruits, military training involves a high amount of physical activity, which can be a new experience for most of the recruits. Therefore, it is important to start physical training during basic training (8 weeks) and in fitness matched groups to ensure that all recruits are able to improve their physical performance in the beginning of their service and also to build a solid platform to increased physical training after the basic training period (Hofstetter, Mäder, & Wyss, 2012; Santtila, Pihlainen, Viskari, & Kyröläinen, 2015; Sporis et al., 2014). Despite technological advances and lighter material, the weight carried by a warfighter has also increased. This is mainly due to the requirement to carry more pieces of equipment which are also heavier, for example, body armors and radios (Drain, Billing, Neesham‐Smith, & Aisbett, 2016; Kraemer & Ratamess, 2005). In addition, the type of tasks a soldier needs to perform has moved towards more explosive type of tasks, for example, close quarters combat. Thus, higher physical requirements combined with the decrease in physical fitness have led to a situation where physical training has to be carefully and systematically tailored to meet the needs of the basic training. When designing a training plan for warfighters, it is important to consider the factors emphasizing the task requirements or performance goals (Nindl et al., 2013). A need for progression, variation, and specificity in training stimulus are the most important factors when considering the development of maximal or explosive strength and maximal aerobic capacity (Friedl et al., 2015; Kyröläinen et al., 2018). Daily military training itself can be short of variety, which may not improve physical performance in an optimal way. For professional warfighters, it is important to have training programs that lead them to reach, as well as to maintain the required level for physical performance (Klien, Hjelvang, Dall, Kruse, & Nordsborg, 2015).

Prolonged military field training has been shown to lead to a decrease in maximal strength and power (Nindl et al., 2002, 2007). Hypertrophic strength training can improve muscular strength by increasing muscle size, while during maximal and explosive strength training gains in strength and power are obtained to a greater extent also by neural factors, for example, increased activation of the agonist muscles (Häkkinen, 1994; Häkkinen et al., 1998). The military environment creates a special platform for strength development because endurance training is always integrated to warfighters’ training programs (Friedl et al., 2015). The effects of military task‐specific training on strength and maximal short duration anaerobic performance of a warfighter has not been investigated. Load carriage studies have shown that added strength training can increase the warfighters’ capability to carry load (Knapik et al., 2004). Whether the same result can be obtained by task‐specific training is unknown, which includes exercises that the soldiers would do in the actual battlefield situation (e.g., loaded sprinting, casualty drags, crawling, climbing or jumping over obstacles, and carrying different objects), is unknown.

Measurement of serum hormone concentrations can be useful in evaluating the effects of different training programs and physiological changes in soldiers’ bodies. Changes in serum hormone concentrations have been found to take place during military field training (MFT). Serum testosterone (TES) is responsible for promoting muscle and bone mass, and has been reported to decrease during MFT by 24%–49% (Alemany et al., 2008; Nindl et al., 2006). Cortisol is released in response to psychological and physical stress, and it has widely been measured during MFT. It has been shown to increase during MFT by 32% (Friedl et al., 2000; Nindl et al., 2007). The TES/COR ratio has been shown to decrease after a week of overreaching endurance training (Adlercreutz et al., 1986) and at the end of a prolonged strength training period (Häkkinen, Pakarinen, Alen, & Komi, 1985), as well as during an intensive high‐volume strength training week in weightlifters (Häkkinen, Pakarinen, Alen, Kauhanen, & Komi, 1988). Similar observations have been made in warfighters (Booth et al., 2006; Fortes et al., 2011). The TES/COR ratio has also been used to study strength training and total physical load in conscripts (Santtila, Kyröläinen, & Häkkinen, 2009; Tanskanen et al., 2011). Added strength training during basic military training (BMT) can also lead to an increase in cortisol concentrations (Santtila et al., 2009). Sex hormone‐binding globulin (SHBG) concentration can increase during 8 weeks of BMT (Nindl et al., 2007), and SHBG is known to inhibit the function of TES. Insulin‐like growth factor‐1 (IGF‐1) has been shown to serve as a potential biomarker for health, fitness, and training status (Nindl et al., 2017). Physical strain and energy deficit have been shown to increase IGF‐1 concentration during MFT (Friedl et al., 2015; Nindl et al., 2002; Vaara, Kokko, Isoranta, & Kyröläinen, 2015). An 8‐week military strength training program can result in an initial increase in IGF‐1 concentration, but these values have then decreased back to existing prevalues after training and consequently no increase between the PRE and POST values have been observed (Nindl et al., 2017).

The purpose of this study was to investigate the effects of two different training programs on strength and power performance and serum hormone concentrations in conscripts during 12 weeks implemented after the basic training period. It was hypothesized that task‐specific training is, at least, as good as strength training, and both of them would improve strength and power performance more than normal military physical training.

## 2 METHODS

### 2.1 Subjects

In all, 104 male conscripts, who were conducting their mandatory military service and had completed their basic training successfully, volunteered as participants for the present study. In the beginning, the three groups had from 33 to 38 participants each, but only 51 of the total number of subjects completed all the measurements during the 12‐week study period (Table 1) leaving the following number of participants who were included in the study as follows: TS (*n* = 19), ST (*n* = 20), and CON (*n* = 12). Reasons for the missed measurements were their overlap with the military training schedule and health‐related problems such as flu or musculoskeletal injury that prevented some participants to either attend the MID or POST testing sessions, thus their data were removed from the analysis. The participants were 18‐ to 22‐year‐old male conscripts. The mean (±*SD*) age was 20 ± 1 years, height 180 ± 6 cm, body mass 72.4 ± 8.8 kg, and body mass index 22.3 ± 2.2 kg/m^2^. The present study was conducted according to the provisions of the Declaration of Helsinki and ethical approval was granted by the Ethical Committee of the University of Jyväskylä. The study was also approved by the Finnish Defence Forces. All the conscripts were informed of the experimental design, and the benefits and possible risks that could be associated with the study prior to signing an informed consent document to participate in the study.

**Table 1:** Mean (±*SD*) age, height, body mass, and BMI in the study groups

Variable	Task specific	Strength	Control
	(*n* = 19)	(*n* = 20)	(*n* = 12)
Age (yrs.)	19.5 (0.8)	19.5 (0.7)	19.6 (0.8)
Height (cm)	180.1 (6.6)	181.5 (6.0)	179.5 (5.4)
Body mass (kg)	74.6 (10.4)	72.3 (7.5)	70.3 (10.2)
BMI (kg·m^−2^)	23.0 (2.2)	22.0 (1.7)	22.4 (2.9)

Abbreviation: BMI, body mass index.

### 2.2 Experimental design

A 12‐week training period was implemented after the basic training period. Body composition, blood samples, and physical performance measurements were repeated three times during the study, in the 10th week (PRE), 16th (MID), and in the 21st week (POST) of their service. On the test days, body composition was measured and blood samples were taken before breakfast. After the breakfast, a series of physical performance tests were performed.

Training programs were designed to be easy to perform during normal weekly physical training. The subjects were all from the same infantry company, and they were divided into three training groups as follows: soldier task‐specific training group (TS), strength‐training group (ST), and control group (CON). Each group was randomly selected platoon from the infantry company and each platoon had a total of 18 training sessions during the 12‐week study with their instructor according to the specific training program. There were some weeks when the conscripts were carrying out MFT when normal physical conditioning was not possible to perform. The number of training sessions varied from one to three per week. Training was regular and periodized, especially, during the first part of the study but during the second part, several field‐training weeks made the conditioning more challenging.

### 2.3 Measurements

Physical testing took place three times during the study (PRE, MID, and POST). Baseline (PRE) testing was made on week 1, MID testing on week 7, and POST testing on week 12 of the study. The testing started in the morning with body composition measurements and blood samples. After the breakfast, the subjects performed muscular power, strength, and muscle endurance tests. Every training group completed their tests during the same day, and the testing was conducted using the same protocol in all measurement points.

#### 2.3.1 Body composition

Body composition characteristics were measured in the morning after an overnight fast between 06:00 and 07:00. The subjects were instructed not to eat anything after their evening meal, which was around 19:00, but they were allowed to drink normally. Body mass (BM), skeletal muscle mass (SMM), fat mass (FM), and fat percentage (FAT%) were measured by using the segmental multi‐frequency bioimpedance analysis assessment (BIA) (InBody 720, Biospace Co Ltd, Seoul, South Korea). The BIA estimates of body composition have shown to highly correlate with the dual‐energy X‐ray absorptiometry (DXA) method (*r* = 0.82–0.95) (Sillanpää et al., 2014).

#### 2.3.2 Serum hormone concentrations

Venous blood samples were drawn three times into Vacutainer® gel tube from the antecubital vein after an overnight fast between 06:30 and 07:30 to analyze serum concentrations of testosterone (TES), cortisol (COR), sex hormone binding globulin (SHBG), IGF‐1, and insulin‐like growth factor binding protein‐3 (IGFBP‐3) (Siemens Immulite 2000 XPI, Siemens Healthcare, USA). The sensitivity and interassay coefficients of variance for these assays were 0.5 nmol/l and 8.2% for TES, 5.5 nmol/l and 7.9% for COR, 0.02 nmol/l and 5.2% for SHBG, 2.6 nmol/l and 7.1% for IGF‐1, and 0.3 nmol/l and 8.4% for IGFBP‐3. The samples were centrifuged (Megafire 1.0 R Heraeus, DJB Lab Care, Germany) after 30 min at 2000 g for 10 min, frozen, and transported to the laboratory for later analysis.

#### 2.3.3 Strength and power performance

All participants were familiarized with all the assessments either due to previous training or before the measurements. Maximal isometric force of the upper (MVCupper) and lower (MVClower) extremities were measured bilaterally in a sitting position. The measurements were conducted using an electromechanical dynamometer manufactured by the University of Jyväskylä, Jyväskylä, Finland. The knee and hip angles were set to 107° and 110° (Häkkinen et al., 1998) in the horizontal leg press position. The knee angle was measured using the trochanter major and the lateral malleolus as reference points. Subjects were told to ensure that they keep contact with the seat and the backrest during the performance. During the measurements of upper extremities, the equipment was adjusted for each subject to their sitting position with their feet flat on the floor, the arms were parallel to the floor, and the elbow angle was 90°. The test was performed by pushing the bar horizontally. The subjects were given one trial attempt before the two actual test trials were conducted for both leg press and bench press with a minimum of 60 s for recovery. In both tests, the subjects were instructed to produce maximal force as fast as possible. During all trials, the testing personnel encouraged them vocally during the maximal effort. The best performances with regard to maximal force output in both tests were selected for further analysis.

Countermovement jump (CMJ) was performed on a contact mat (Newtest, Oulu, Finland). Subjects were instructed to keep their arms on their hips while performing the jump. Subjects started their maximal vertical jumps from a standing position, hands on their hips. The lowest knee angle was instructed to be 90°. All the subjects had three jump attempts. Flight time from contact to contact was used to calculate the jumping height for each jump (Bosco, Luhtanen, & Komi, 1983).

A 6‐s maximal anaerobic power cycle ergometer test (Wattbike Ltd) was used to measure maximal power in the lower extremities. The 6‐s test has a seated stationary start with the dominant leg initiating the first down‐stroke. Before the test, each subject's weight was inserted into the test bikes computer and air and magnetic resistance were set accordingly. The test started following a 5‐s countdown followed by verbal command. The completion of the test was indicated also with another verbal command (Herbert, Sculthorpe, Baker, & Grace, 2015).

Standing long jump (SLJ) was used to measure explosive force production of the lower extremities (Bosco et al., 1983). The jumps were performed on a 10‐millimeter‐thick plastic mattress designed for the purpose (Fysioline Co). Conscripts were given instructions of a proper technique, and two to three warm‐up trials before the actual three test attempts. The jumps were performed from a standing position, feet at pelvis to shoulder width. Take off was assisted by extension of the hip and swinging of the arms. The landing was performed bilaterally and falling backwards led to the disqualification of the attempt. The result of the best jump was measured in centimeters from the shortest distance from the landing point to the starting line.

Muscle endurance capacity of the trunk and upper extremities was measured using sit‐up and push‐ups tests. A test supervisor showed the proper technique for both performances before the tests. Sit‐ups were used to measure abdominal and hip flexor performance (Viljanen, Viitasalo, & Kujala, 1991). In the starting position, the conscript laid on his back, while the legs were supported from the ankles by an assistant. The knee angle was 90°, elbows pointing upwards, and fingers crossed behind the head. A successful repetition was credited when the conscript lifted his upper body from the starting position and brought elbows to the knee‐level. The result was the number of successful repetitions during 60 s. Push‐ups were used to measure performance of the arm and shoulder extensor muscles (ACSM, 2000). Before taking a starting position, the conscript laid face down on the floor, feet parallel at pelvis to shoulder width, and hands positioned so that the thumbs could reach the shoulders while other fingers pointing forward. Before the initiation of the test, the conscripts were instructed to extend their arms to the starting position and keep the feet, trunk, and shoulders in the same line throughout the test. One successful repetition was counted when the soldier lowered his torso by flexing arms to an elbow angle of 90° and returned to the starting position by extending his arms. The result was the number of successful repetitions during 60 s.

### 2.4 Training protocols

The total amount of instructed physical training sessions performed by the subjects was 12 during the first 6 weeks and 6 during the latter 6 weeks of the study (Table 2). They also conducted their normal military training every day, which consisted of road marches, shooting exercises, and other task‐specific exercises. Variation of the instructed physical training program was due to MFTs that their schedule had. During those weeks, the conscripts were not able to do any extra physical training. When planning the training program for our study, we tried to consider these factors, as well as possible. The most demanding physical training sessions were done during the lightest military training weeks. All the groups participated in the instructed physical training, but the content of training varied between the groups. All groups performed 10 min active warm‐up consisting of muscular activation before starting the actual exercise. The total length of one training session was 60 min in all groups.

**Table 2:** Design of the training protocols in the study groups

Session	Task‐specific group			Strength group			Control group		
	1	2	3	1	2	3	1	2	3
Week 1	PRE tests			PRE tests			PRE tests		
Week 2	2x3x6s	2x3x30s	3x6s; 3x30s	4x3x15x10−30%	4x3x15x10−30%	4x3x10x40−60%	Military PE	Military PE	Military PE
Week 3	2x4x6s	2x4x30s	4x6s; 4x30s	4x3x10x40−60%	BWT x 5 exercises	BWT x 5 exercises	Military PE	Military PE	Military PE
Week 4	3x3x6s	3x3x45s	4x6s; 4x45s	6x3x6x20−40%	4x3x3−5x70−90%	4x3x8x50−75%	Military PE	Military PE	Military PE
Week 5	3x3x6s	3x3x45s	4x6s; 4x45s	6x3x6x20−40%	4x3x3−5x70−90%	4x3x12x50−75%	Military PE	Military PE	Military PE
Week 6	Military field training			Military field training			Military field training		
Week 7	MID tests			MID TESTS			MID tests		
Week 8	3x3x6s	4x45s	3x3x60s	BWT x 5 exercises	BWT x 5 exercises	4x3x3−5x70−90%	Military PE	Military PE	Military PE
Week 9	Military field training			Military field training			Military field training		
Week 10	3x4x6s	3x6s; 3x60s	3x4x6s	5x3x4−6x70−90%	6x3x4−6x20−40%	5x2x4−6x70−90%	Military PE	Military PE	Military PE
Week 11	Military field training			Military field training			Military field training		
Week 12	POST tests			POST tests			POST tests		

#### 2.4.1 Task‐Specific group (TS)

The task‐specific group performed basic infantry‐based exercises with the full combat gear (27 kg), such as sprints, crawling, casualty drag, and climbing over obstacles. The exercises focused on anaerobic or speed. The TS exercises performed with speed emphasis were conducted for 6 s, performed with maximal effort and a 2‐min recovery time between the sets. The TS exercises performed with anaerobic emphasis were conducted for 30–60 s for each repetition, with 75%–90% of maximal effort and a 1‐min recovery time between the sets (Table 2).

#### 2.4.2 Strength group (ST)

The strength group had a nonlinear training program, which included muscular endurance, hypertrophic, and maximal strength and power training. Strength training started with lower intensity and higher repetitions (10–15 repetitions with 10%–60% of 1RM), but soon turned into hypertrophic (8–12 repetitions with 50%–75% of 1RM), maximal strength (3–6 repetitions with 70%–90% of 1RM) or power (4–6 repetitions with 20%–40% of 1RM) workouts (Table 2). There were four to five different exercises (e.g., squat, deadlift, bench press and different push, and pull exercises for upper body) in each session and three to six sets per each exercise.

#### 2.4.3 Control group (CON)

The control group trained according to the normal Finnish military physical education guidelines, which included muscular endurance training, ball games, and endurance training. A normal training session included, for example, circuit training with body weight, running with constant pace or playing basketball, football, or floorball. The total amount of their training was the same compared to the other groups (Table 2).

### 2.5 Statistical analysis

The data for the present study were analyzed using SPSS Statistics 24 (IBM SPSS 24.0 Chicago, Illinois). All data were checked for normality. For calculating means, 95% confidence intervals, standard deviations (±*SD*), and Pearson product moment correlation coefficients conventional statistics were used. Probability adjusted *t* tests were used for pairwise comparisons when appropriate. The effect size was calculated as the mean difference between the PRE, MID, and POST measurement values divided by the standard deviation of the values (0.2 = small; 0,5 = medium; 0,8 = large effect (Cohen, 1988). A general linear model, with repeated measures ANOVA with group as a fixed factor, was used to analyze time * group interaction and the differences between the different measuring points. Bivariate correlation was used to assess associations between the changes in time. The *p* < .05 criterion was used for establishing the statistical significance.

## 3 RESULTS

### 3.1 Body composition

Significant increases from PRE to POST and MID to POST were found in body mass in ST (1.5 ± 2.8% and 0.8 ± 1.7%; Table 3). In addition, significant increases were found in SMM between the PRE and MID measurement points in the TS (1.7 ± 3.0%) and ST (1.0 ± 1.9%) groups. A significant drop in FM was observed in the TS (−9.5 ± 13.5%) group between the PRE and MID measurements, but FM (11.1 ± 21.7%) recovered almost back to the PRE values from the MID to POST measurements. No significant differences were observed between the groups in any measurement points.

**Table 3:** Mean (±*SD*) values of body composition in the study groups

	Group	PRE		MID		POST		|Effect size|		
		Mean (±*SD*)	95% CI	Mean (±*SD*)	95% CI	Mean (±*SD*)	95% CI	1 versus 2	2 versus 3	1 versus 3
BM (kg)	TS	74.6 ± 10.4	69.6; 79.6	74.3 ± 9.7	69.7; 79.0	74.8 ± 9.4	70.2; 79.3	0.03	0.05	0.02
	ST	72.3 ± 7.7	68.5; 76.0	72.7 ± 7.5	69.1; 76.3	73.3 ± 7.9 ^#, §^	69.5; 77.1	0.05	0.08	0.13
	CON	70.3 ± 10.2	63.8; 76.7	69.8 ± 9.0	64.1; 75,5	70.1 ± 8.3	64.8; 75.3	0.05	0.03	0.02
SMM (kg)	TS	36.7 ± 4.8	34.4; 39.0	37.3 ± 4.7^*^	35.0; 39.6	37.1 ± 4.8^#^	34.8; 39.4	0.12	0.04	0.08
	ST	37.0 ± 4.1	35.1; 38.9	37.4 ± 4.0^*^	35.5; 39.3	37.5 ± 4.1	35.5; 39.4	0.10	0.02	0.12
	CON	34.7 ± 4.3	31.9; 37.4	34.8 ± 4.4	32.0; 37.5	34.8 ± 4.5	31.9; 37.6	0.02	0.00	0.02
FM (kg)	TS	10.1 ± 4.6	7.9; 12.2	8.9 ± 3.7^*^	7.1; 10.7	9.7 ± 3.7^§§^	7.9; 11.5	0.28	0.21	0.09
	ST	7.3 ± 2.8	6.0; 8.6	7.2 ± 2.9	5.9; 8.6	7.9 ± 3.1^§§§^	6.4; 9.3	0.03	0.23	0.20
	CON	9.2 ± 5.1	6.0; 12.4	8.7 ± 4.3	6.0; 11.4	8.9 ± 3.7	6.6; 11.3	0.10	0.05	0.07
FAT%	TS	13.3 ± 4.7	11.0; 15.5	11.9 ± 3.9^*^	10.0; 13.8	12.9 ± 4.1^§§^	10.9; 14.9	0.32	0.24	0.09
	ST	10.1 ± 3.5	8.4; 11.7	9.9 ± 3.5	8.2; 11.5	10.6 ± 3.6^§§§^	8.9; 12.3	0.06	0.19	0.14
	CON	12.6 ± 5.7	9.0; 16.3	12.3 ± 5.4	8.9; 15.7	12.7 ± 4.6	9.7; 15.6	0.05	0.08	0.02

Abbreviations: BM, body mass; CON, control; FAT%, fat percentage; FM, fat mass; SMM, skeletal muscle mass; ST, strength; TS, task‐specific. * = PRE to MID values *=*p* < .05; # = PRE to POST values, #=*p* < .05, MID to POST values §= *p* < .05, §§=*p* < .01, §§§<0.001.

### 3.2 Strength and power performance

In the strength and power performances most of the significant increases took place in the TS and ST groups, both during the first part and after the entire experimental training period (Table 4). Isometric leg press force increased significantly by 7.9 ± 12.2% (*p* < .05) for TS and by 7.1 ± 12.6% (*p* < .05) for ST between the PRE and MID measurements. A significant increase in leg press force was also observed in TS 8.1 ± 12.4% (*p* < .05) and ST 12.3 ± 15.3% (*p* < .01) between the PRE and POST measurements. Isometric horizontal bench press force increased significantly by 4.7 ± 6.8% (*p* < .01) between the PRE and MID and by 7.1 ± 7.0% (*p* < .01) between the PRE and POST measurements. Maximal power in the 6‐s cycling test increased significantly between PRE and MID by 4.4 ± 6.9% (*p* < .05) in TS and 4.1 ± 4.8% (*p* < .01) in ST, as well as between the PRE and POST measurements in TS 6.0 ± 6.3% (*p* < .05), in ST 4.1 ± 5.3% (*p* < .01) and in CON 5.3 ± 5.5% (*p* < .01). In push‐ups, there were significant increases between the PRE and MID measurements in TS 26.5 ± 33.4 (*p* < .01) and in ST 14.0 ± 13.1 (*p* < .05) groups. Between PRE and POST, the CON group improved significantly 31.0 ± 26.9 (*p* < .01) its push‐ups performance. No significant differences were observed between the groups in the PRE measurements. The CON group differed significantly from the TS and ST groups in lower body strength in the MID and POST measurements and also in standing long jump in the POST measurement point.

**Table 4:** Mean (±*SD*) strength and power performance values in the study groups

	Group	PRE		MID		POST		|Effect size|		
		Mean (±*SD*)	95% CI	Mean (±*SD*)	95% CI	Mean (±*SD*)	95% CI	1 versus 2	2 versus 3	1 versus 3
CMJ (cm)	TS	30 ± 5	27; 32	29 ± 5	27; 31	31 ± 4^**, ###^	29; 33	0.20	0.43	0.22
	ST	32 ± 6	29; 35	33 ± 5	30; 35	34 ± 5	32; 36	0.18	0.20	0.35
	CON	31 ± 6	28; 35	30 ± 7^*^	25; 34	31 ± 6	28; 35	0.15	0.15	0.00
Power Max (w)	TS	1,038 ± 153	964; 1,112	1,075 ± 152^*^	1,002; 1,149	1,096 ± 141^*, ##^	1,028; 1,164	0.24	0.14	0.39
	ST	1,070 ± 112	1,012; 1,119	1,119 ± 120^**^	1,054; 1,159	1,120 ± 126^**^	1,051; 1,165	0.41	0.01	0.41
	CON	952 ± 104	896; 1,040	992 ± 136	905; 1,078	1,017 ± 139^**^	928; 1,105	0.32	0.18	0.51
Upper body strength (kg)	TS	79 ± 16	71; 86	77 ± 14	70; 83	80 ± 13^###^	74; 87	0.13	0.22	0.07
	ST	77 ± 15	70; 84	82 ± 16^**^	72; 88	82 ± 17^**^	74; 90	0.32	0.00	0.31
	CON	72 ± 15	64; 83	73 ± 12	70; 83	76 ± 15	65; 86	0.07	0.21	0.26
Lower body strength (kg)	TS	240 ± 46	219; 263	255 ± 49^*^	232; 279	263 ± 48^**^	240; 286	0.32	0.16	0.48
	ST	227 ± 46	205; 248	240 ± 37^*^	222; 257	240 ± 38^*^	222; 258	0.31	0.00	0.30
	CON	214 ± 51	182; 247	220 ± 61 μ	181; 259	222 ± 60 μ	184; 261	0.10	0.03	0.14
Standing long jump (cm)	TS	216 ± 25	204; 228	215 ± 21	205; 225	216 ± 21	205; 226	0.04	0.05	0.00
	ST	228 ± 17	220; 237	228 ± 18	218; 233	229 ± 17	220; 235	0.00	0.06	0.06
	CON	214 ± 32	194; 234	210 ± 28	192; 228	210 ± 31μ	190; 229	0.13	0.00	0.12
Sit‐ups (reps/min)	TS	41 ± 10	36; 45	42 ± 9	37; 46	40 ± 12	34; 46	0.10	0.18	0.09
	ST	44 ± 9	39; 48	45 ± 8	40; 48	45 ± 9	40; 48	0.12	0.00	0.11
	CON	41 ± 7	36; 46	43 ± 8	38; 48	42 ± 7	37; 47	0.26	0.13	0.14
Push‐Ups (reps/min)	TS	34 ± 13	28; 40	40 ± 14^**^	33; 47	38 ± 15	31; 45	0.43	0.13	0.28
	ST	38 ± 12	32; 44	43 ± 13^*^	36; 48	41 ± 14	34; 47	0.39	0.23	0.15
	CON	35 ± 14	25; 45	41 ± 15	29; 50	44 ± 16^**^	33; 54	0.40	0.19	0.58

Abbreviations: CMJ, counter measurement jump; CON, control; Lower body strength in maximal isometric action; Power ave, average 6 s power cycling; Power max, maximal 6 s power cycling; ST, strength; TS, task‐specific; Upper body strength in maximal isometric action. * = PRE to MID values *=*p* < .05; # = PRE to POST values, #=*p* < .05, ##=*p* < .01, ###<0.001, MID to POST values §= *p* < .05, §§=*p* < .01, §§§<0.001; between the groups, μ = *p*<.05.

### 3.3 Changes in serum hormone concentrations.

**Figure 1:**
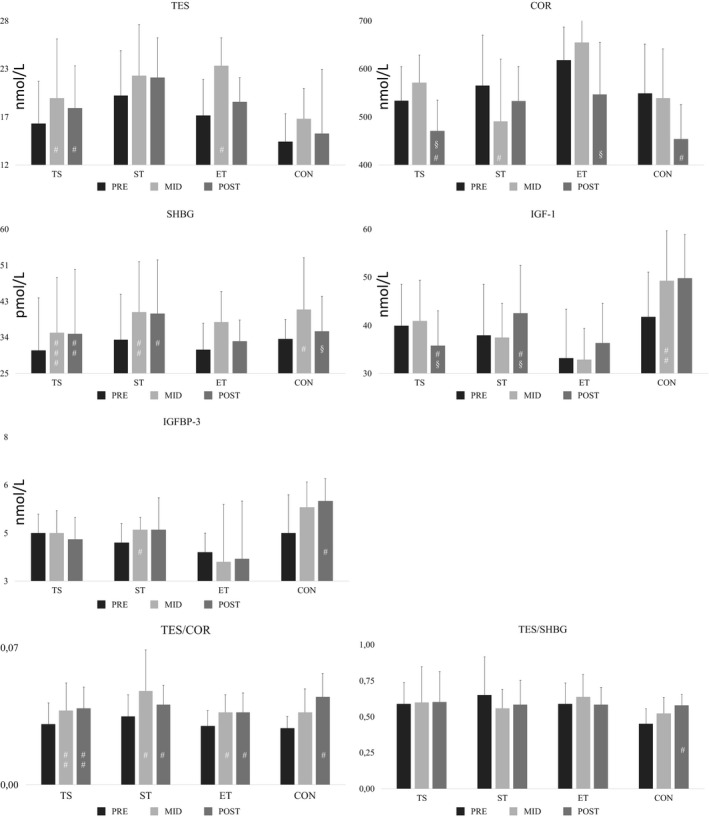
Mean (±SD) serum hormonal concentrations in the groups. COR, cortisol; CON, control; IGF‐1 insulin‐like growth factor 1; IGFBP‐3, insulin‐like growth factor binding protein 3; SHBG, sex‐hormone binding globulin; ST, strength; TES, testosterone; TES/COR, testosterone – cortisol ratio; TES/SHBG, testosterone – sex‐hormone binding globulin; TS, task specific. Within the groups; #=*p* < .05, ##=*p* < .01, ###=*p* < .001, compared to PRE value; §=*p* < .05, compared to MID value.

Serum TES concentration increased significantly in TS between the PRE and MID (16.8 ± 33.9%) and PRE and POST (11.2 ± 16.7%) measurements (Figure 1). Serum COR concentrations decreased in TS between the MID and POST (−7.8 ± 10.9%) and PRE and POST (−11.0 ± 14.3%) measurements. In CON, a decrease in COR concentration was found between PRE and POST (−16.3 ± 14.8%). Serum SHBG concentrations increased both in the TS and ST groups between PRE and MID (18.2 ± 19.6% for TS and 24.9 ± 30.3 for ST) and between PRE and POST (11.0 ± 17.4% for TS and 23.5 ± 38.2% for ST). In CON SHBG first increased significantly (20.0 ± 32.9%) between PRE and MID and then decreased significantly (−5.8 ± 25.2%) between MID and POST. IGF‐1 concentration decreased in TS between MID and POST (−11.1 ± 15.1%) and PRE and POST (−7.7 ± 16.0%). The CON group showed significant increases in IGF‐1 between PRE and MID (10.5 ± 10.9%). IGFBP‐3 concentration increased significantly in ST between PRE and MID (9.8 ± 14.4%) and in CON between PRE and POST (29.5 ± 46.2%). The TES/COR‐ratio increased significantly in all groups between PRE and MID and PRE and POST, while a significant change was only observed between PRE and POST in CON.

## 4 DISCUSSION

The main objective of this study was to find out how different physical training modes influence warfighter's strength and power performances and serum hormone concentrations during the 12‐week conscript training period. All three groups conducted the same volume of physical training, and only the content of the training mode varied between the groups. The main findings showed that both task‐specific and strength training programs increased maximal strength and power performances to a greater degree compared to normal military physical training. The findings support our hypothesis of the need for strength and power related training in soldiers. Daily military training may also have a great impact on the development of different physical characteristics. In the present study, physical training was highly compromised by military field training and only minor improvements took place in the latter part of the study.

In the previous study with conscripts (Santtila, Häkkinen, Karavirta, & Kyröläinen, 2008), it has been shown that basic military training leads to increases in physical performance including both endurance and strength capability. Similar results have been reported also during US Army Basic Training (Hendrickson et al., 2010; Knapik et al., 2001). Increases in strength and power have also been reported in previously trained warfighters (Abt et al., 2016; Lester et al., 2014; Solberg et al., 2015). The present TS and ST groups showed the highest increase in strength and power measurements, both between the PRE and MID and PRE and POST measurement points. The training was not interrupted by tactical field training that much during the first weeks of the study as during the last weeks. The plateau observed between the MID and POST measurement points was likely due to the decreased amount of controlled training and the increased amount of field training. Low volume of training sessions (1 to 2) per week may not be enough to lead to large changes in body composition or strength/power gains. In the present study, the most improvement was observed during the first 6 weeks when the amount of training sessions was 2–3 per week. During the last 6 weeks not much difference between the groups was observed, because of the high amount of military field training that made it difficult to carry out physical training as planned. The present CON group had also some improvements in strength and power performances, but not as much as in the TS and ST groups. It is also possible that the greater volume of endurance training in the CON group might have also interfered with their strength and power development compared to the TS and ST groups. Due to the present study design, we could not evaluate possible changes in maximal aerobic performance in any of the present groups. The similarity of the gains in the ST and TS groups were probably mostly because of the increased training stimuli for upper and lower body muscles. The training in the TS group with extra weight was similar to that of the weight training in the ST group with regard to the intensity and duration.

In previous studies, decreases in serum hormonal concentrations have been reported as a response to physical exertion combined with energy and sleep deprivation (Alemany et al., 2008; Friedl et al., 2000; Kyröläinen et al., 2008; Nindl et al., 2007; Richmond et al., 2014). Intensive field training can lead to drastic declines in total TES and IGF‐1 concentrations (Nindl et al., 2007). Friedl et al. (2000) showed that significant decreases in TES and IGF‐1 concentrations occurred after the 8‐week military field training period with increases in SHBG and COR concentrations. The present study did not find such drastic changes. The TES and IGF‐1 levels were either unaltered or increased during the study. SHBG seemed to increase to some extent, and COR remained at the same level or even decreased during the study. These results suggest that the conscripts were not physically overtrained during the present study. Most of the changes in the measured serum concentrations may refer to an improved anabolic state. Therefore, it seems that the conscripts were not overtrained during their latter part of their conscript service, although the increases in the present strength and power performance variables remained minor between the MID and POST measurements.

Total TES concentration has been shown to lead to an acute increase after resistance exercise in men (Hakkinen & Pakarinen, 1993; Kraemer & Ratamess, 2005). Tremblay, Copeland, and Van Helder (2004) reported that the acute elevation in TES was greater in strength trained men than in endurance trained. This could suggest that strength training could have beneficial periodical (Häkkinen, Pakarinen, Alen, Kauhanen, & Komi, 1987; Häkkinen et al., 1985, 1988) adaptations in TES as observed in strength trained athletes (Häkkinen et al., 1987, 1988). In the present study, the increases in TES took place during the first 6 weeks but this was not replicated during the last 6 weeks of the intervention (Figure 1). Concurrent military training could have contributed to the present findings. Similar acute increases have been found in SHBG concentrations, but not in resting values in longer‐term studies (Kraemer & Ratamess, 2005). There was a significant increase in SHBG in all the groups from PRE to MID in the present study but from MID to POST, SHBG concentrations remained statistically unaltered. COR concentrations have been shown to increase acutely, but no consistent pattern has been found in long‐term training stress (Kraemer & Ratamess, 2005). COR concentrations decreased in all groups from the PRE to POST measurements. This resulted in a rise in the TES/COR‐ratio, which could indicate that the conscripts were unlikely overtrained. Resting IGF‐1 concentration has been shown to be higher in resistance‐trained than untrained men. It appears that the volume and training intensity are important factors for chronic IGF‐1 adaptations (Kraemer & Ratamess, 2005). In the present study, the increases in IGF‐1 were observed in the CON and ST groups. The same was found also with regard to IGFBP‐3 concentrations. These findings indicate that different types of concurrent training can elevate anabolic hormone concentrations and contribute to an increase in muscle mass as observed in the present TS and ST groups. It is important to have intensive strength training during military training, while attempting to create both acute and chronic positive hormonal changes, as well as increased maximal strength and power of the trained muscles.

During the present study the conscripts were completing their military service, which involves a high volume of low aerobic type of activity with constant load carriage. Because of physical and tactical demands of their training, this type of activity is unavoidable. The regular day‐to‐day military training schedule did not allow most of the time for periods of rest after field exercises or physical training activities. That is why, it is likely that this concurrent type of training had at least some effect on the physical performance of the subjects, but the purpose was to determine what kind of training might be most beneficial during normal military service. In addition, MFT may have created inconsistent training responses during the second part of the study. Also, the MID and POST measurements were done after the MFT week due to the tight training schedule. The present study was limited by the small number of subjects per group, which may have reduced the generalizability of the present findings. The training program for the ST and TS groups were quite different from that taking place during the basic training. All exercises were familiar for most of the subjects, but better familiarization might lead to better results in the future. Also, the effectiveness of military task‐specific training that TS group did should be studied more in detail in the future. We had a task‐specific military simulation test in our measurements, but we did not include those results as we wanted to concentrate on changes in body composition, strength/power, and hormonal measurements. More periodization between military training and physical training is needed in the future to achieve better enhancement of physical performance. In addition, the future research should follow the development of strength and power throughout the conscript service. This would help to develop more optimal long‐term training program for strength and power in a military environment.

In conclusion, training in the TS and ST groups led to greater improvements in muscle strength and power than training in the CON group. Especially, the increases in lower body strength and power, as well as in upper body strength were greater in the TS and ST groups. These findings are important for warfighter performance. In previous studies, the need for the high level of strength has been found to be vital to warfighter (Friedl et al., 2015; Kraemer & Szivak, 2012). Daily military training may consist too much of low‐intensity aerobic work which does not lead to the increase in warfighters’ strength.

## 5 CONCLUSION

By creating variation in the training content during the conscript service, it is possible to make improvements in different components of the physical performance capacity, especially in strength and power, which can be translated to improvement in common tactical movements (i.e., marching, load carriage, and fire and movement drills). With structured, regular, and controlled physical training, the impact on conscripts’ physical performance would be greater than with the current military physical training. However, successful strength and conditioning program for conscripts cannot be built without taking into account the fracture nature of training caused by military‐specific training requirements. It is important in the future that the leadership prioritizes quality‐controlled physical training and supports necessary changes. Strength and task‐specific training can lead to improvements in the battlefield, reduce the risk of overtraining, and prevent injuries caused by excessive running and loaded marching during the traditional military training. Furthermore, in the future, it is important to study the effects of ST and TS after basic training for optimizing the periodization, duration, intensity, and frequency of strength and power training on the development of the physical performance capacity required for the battlefield.
